# Molecular biology of pituitary neuroendocrine tumors

**DOI:** 10.1007/s11060-026-05447-0

**Published:** 2026-02-04

**Authors:** Reilly L. Kidwell, James Trippett, Manish K. Aghi

**Affiliations:** Department of Neurological Surgery, University of California, San Francisco, 505 Parnassus Ave, M-779, San Francisco, CA 94143-0112 USA

**Keywords:** PitNETs, Molecular drivers, Transcription factors, Clinical trials

## Abstract

**Background:**

Pituitary neuroendocrine tumors (PitNETs) represent a heterogeneous group of intracranial neoplasms arising from the anterior pituitary gland. While most tumors are benign, certain subsets can display aggressive behavior marked by invasiveness, treatment resistance, and familial clustering. The current World Health Organization (WHO) classification emphasizes the role of lineage-specific transcription factors in better identifying cell types. However, this methodology is not sufficient to ensure fully accurate prediction of tumor behavior; therefore, new, more in-depth methods are required to improve diagnostic reliability and treatment decision-making.

**Methods:**

A narrative review was carried out to evaluate the literature on PitNET classification schemas and their molecular signatures. Attention was placed on classification research and developments that impact current clinical management.

**Analysis:**

Evidence indicates improvement in the molecular classification of PitNETs, not just from lineage-specific transcription factors, but also from advances in genomic, transcriptomic, and epigenetic profiling. These newer techniques have revealed that PitNETs are driven by a complex interplay of alterations, including somatic mutations, germline predisposition genes, copy number variations, and poorly regulated signaling pathways. Each of these general findings plays a role in influencing tumor behavior, controlling lineage differentiation, and determining response to therapy. These findings indicate the need for integrating molecular characteristics with clinical data to improve risk stratification and guide personalized treatment.

**Conclusion:**

Clinical data combined with molecular classification systems is redefining our understanding of PitNET behavior and improving clinical decision-making by increasing our diagnostic accuracy and advancing our knowledge of individualized patient tumor biology. Continued research and development of comprehensive predictive approaches are necessary to achieve reliable outcome prediction and improve therapeutic decision-making for all patients.

## Introduction

**Fig. 1:**
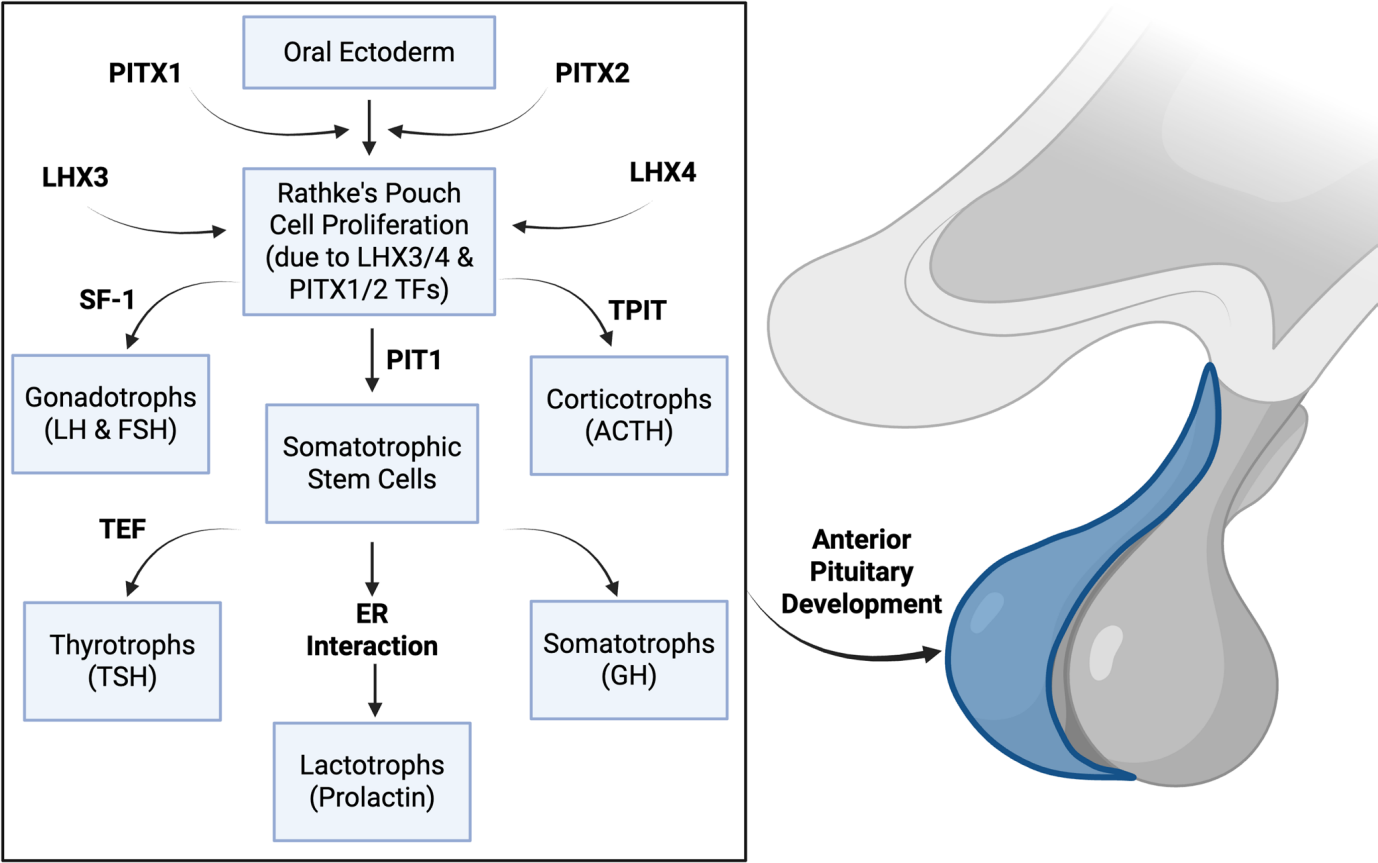
Developmental hierarchy and transcription factor networks guiding anterior pituitary cell differentiation. Outlined here is the developmental progression of the anterior pituitary and the specific TFs that lead to the development of its different cell types. Gonadotrophs, Corticotrophs, and Somatotrophs occur earlier in development due to the transcription factors SF-1, TPIT, and PIT1, respectively. The last cells to develop are Lactotrophs and Thyrotrophs, which form from the common precursor Somatotroph cells. They do this through interactions involving the Estrogen receptor in Lactotrophs and Thyrotroph embryonic factor (TEF)

Key lineage‑specifying transcription factors (TFs) in pituitary development are PIT1, TPIT, and steroidogenic factor‑1 (SF‑1). PIT1 is upregulated in GH, PRL, and TSH‑expressing cells; TPIT, a T‑box factor, marks ACTH cells; and SF‑1 is expressed in gonadotrophic cells (Fig. 1). The development of the pituitary gland involves a complex interplay between dorsal and ventral signaling centers and is tightly regulated. The anterior pituitary (adenohypophysis) arises from the oral ectoderm as Rathke’s pouch, which grows dorsally toward the ventrally positioned diencephalon, the source of the posterior pituitary (neurohypophysis) [1]. Key TFs guiding this development include PITX1 and PITX2, which are essential for initial pouch formation, and LHX3 and LHX4, which promote pituitary cell proliferation and differentiation [2]. The anterior pituitary gland is comprised of six hormone-producing cell types: somatotrophs, lactotrophs, thyrotrophs, corticotrophs, and gonadotrophs [1]. Precursor cells expressing PIT1 can differentiate to form somatotrophs, lactotrophs, or thyrotrophs via interactions with specific receptors (e.g. estrogen receptor) which enhance secretion of specific hormones; SF-1 and TPIT are required for gonadotroph and corticotroph differentiation, respectively [3]. On the ventral side, signaling molecules such as sonic hedgehog (SHH) from the oral ectoderm and bone morphogenic protein 4 (BMP4) and fibroblast growth factors (FGFs) from the infundibulum of the diencephalon coordinate to guide the spatial and temporal development of the gland [4]. Specific disruptions or overactivations of TFs and signaling gradients can lead to congenital pituitary deficiencies and have also been implicated in tumorigenesis.

## Pituitary neuroendocrine tumor classification

Historically, pituitary tumors were classified based primarily on the hormones they secreted and their structural features. In recent years, a growing understanding of pituitary embryogenesis and tumorigenesis has led to a paradigm shift toward a lineage-based molecular classification, emphasizing the TFs that govern cell differentiation within the anterior pituitary. The 2017 World Health Organization (WHO) classification incorporated these lineage markers by stratifying pituitary tumors according to their expression of lineage-specific TFs. This framework previously identified three major cell lineages defined by the TFs TPIT, SF-1, and PIT1. Tumors were accordingly subclassified as corticotroph adenomas (TPIT-positive), gonadotroph adenomas (SF-1-positive), or PIT1-lineage adenomas, which include lactotroph, somatotroph, thyrotroph, and plurihormonal tumors. Tumors lacking expression of any of these markers were termed null cell adenomas [5]. The 2022 WHO update introduced a significant conceptual shift by redefining these tumors as pituitary neuroendocrine tumors (PitNETs), aligning them nomenclaturally with other neuroendocrine neoplasms. This change emphasizes that PitNETs, like other neuroendocrine tumors (NETs), span a biologic spectrum with variable differentiation, potential for recurrence, and rarely, aggressive or metastatic behavior. The update also prioritizes immunohistochemistry as a cornerstone of classification, not only for hormone identification but also for TF profiling and proliferation indices (e.g., Ki-67) [6]. Importantly, while these classification schemes are of interest from a molecular pathology perspective, they do not necessarily predict tumor behavior, guide therapy, or reflect underlying tumor biology [7].

NETs are defined by several features, including the presence of dense core secretory granules, the production of peptide hormones, and the expression of common neuroendocrine markers such as synaptophysin, chromogranin A, neuron-specific enolase (NSE), and INSM1 [8]. In contrast, classic endocrine tumors, such as those from the adrenal cortex or thyroid follicular cells, more commonly feature smooth endoplasmic reticulum to support steroidogenesis, secrete steroid hormones or amines, exhibit few somatic mutations, and express site-specific TFs or cytokeratins (e.g., SF-1 in adrenal tumors, PAX8 in thyroid tumors). While TF–based classification has significantly advanced diagnostic precision, emerging molecular profiling studies have revealed additional layers of heterogeneity within each lineage, underscoring the need to explore the distinct genomic and epigenetic landscapes that define each PitNET subtype. While these molecular markers have not been used in clinical practice to date, research showing their connection to PitNETs could eventually lead to their incorporation into the diagnostic process.

## Epidemiology of pituitary neuroendocrine tumors

According to the Central Brain Tumor Registry of the United States Statistical Report for brain tumors between the years 2016 to 2020, tumors involving the pituitary gland were the second most common tumor of the brain and central nervous system, accounting for 17.2% of all tumors [9]. For each pituitary tumor subtype, calculating the exact incidence is difficult due to variable patient samples and the fact that not all pituitary tumors are treated surgically and therefore lack clear pathology. Nonetheless, in the literature, the most prevalent pituitary tumor subtype is non-functioning pituitary tumors, with prevalence ranging from 22% to over 50% [10–12]. For each subtype, the percentages show high variability; however, the most common hormone-secreting pituitary tumor subtype is prolactinoma, which accounts for roughly 30% to 44% of pituitary tumors treated [10, 13, 14]. 

Looking into resected tumors and breaking them down by their lineage and TF profiles provides greater detail on how common each tumor type is. For example, in one study with over 1000 patients who underwent resection, gonadotroph PitNETs were the highest represented tumor classification with 42.5% of tumors resected. PIT-1-expressing tumors were next, with 29.9% of tumors resected, followed by corticotroph tumors at 17.1%, with null cell tumors making up the smallest percentage at 4.5% of tumors in this patient cohort [13]. Prolactin-secreting tumors are not in this cohort because most can be treated medically and therefore do not require surgical resection [13, 15].

## Molecular insights by subtype

The lineage-based classification of PitNETs does yield some insight to patterns of molecular differentiation. For example, PIT1-positive tumors frequently exhibit widespread DNA hypomethylation and chromosomal instability, features that may contrast with the more epigenetically stable profiles of TPIT or SF-1–lineage tumors. However, recent single-cell RNA sequencing studies challenge the rigid separation of these subtypes by demonstrating that multiple lineage-specific transcriptional programs can coexist within individual tumors, with classification often reflecting the predominant cell population rather than absolute exclusivity [16]. This underscores the importance of moving beyond TF-expression alone to define PitNET behavior.

**Fig. 2:**
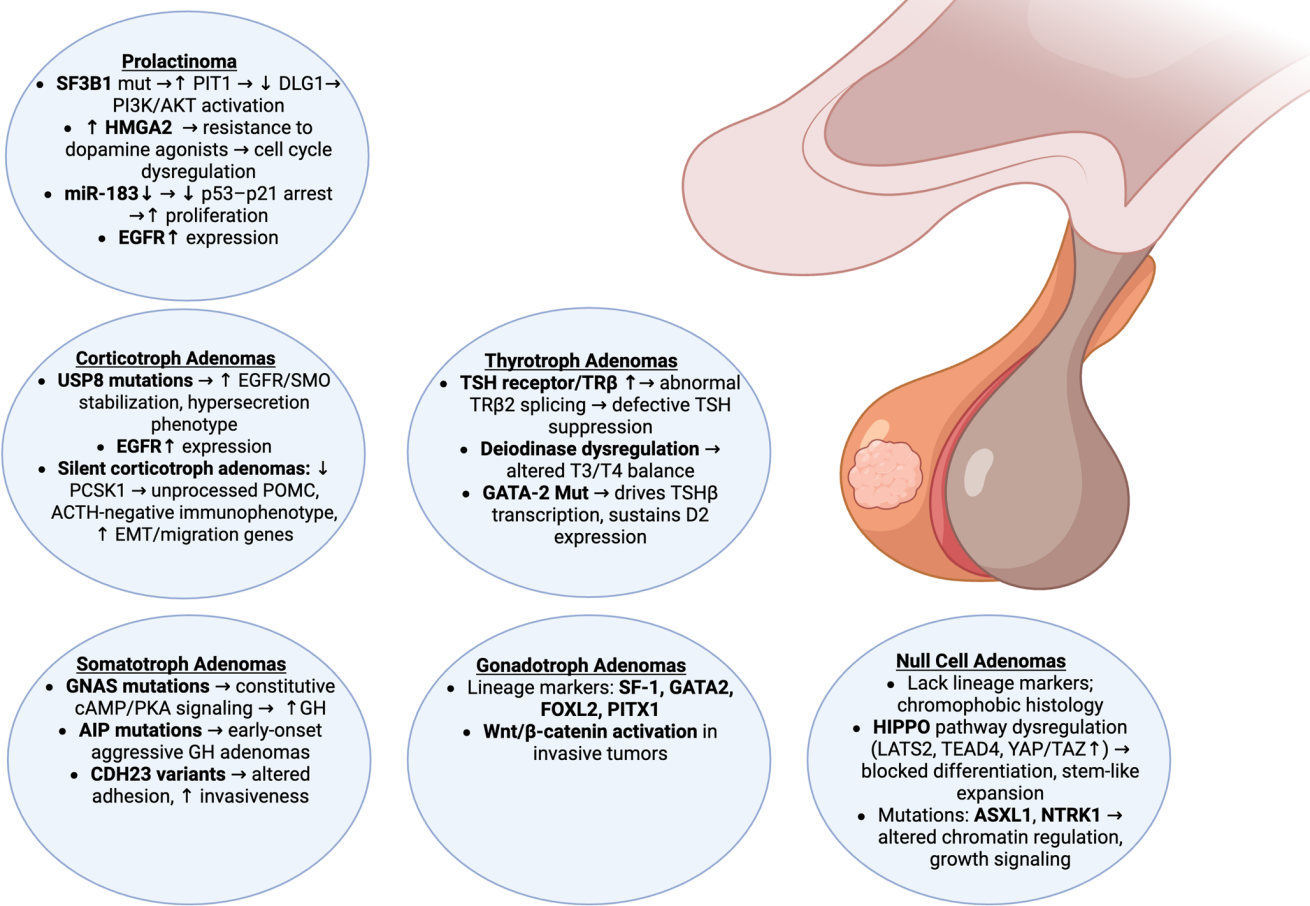
Molecular alterations and key biomarkers across PitNET subtypes. PitNETs display molecular signatures that are distinct and based on their lineage. This outline demonstrates the key markers present in each subtype and describes the primary consequence of each genetic alteration and marker expression. The physician can utilize each PitNET’s specific aberration and marker expression to better characterize the tumor’s possible aggressiveness and help clinician-researchers discover more personalized treatment in the future based on individual patient presentation

Chromosomal instability and somatic mutation burden have long been recognized as hallmarks of cancer. In PitNETs, these genomic alterations appear to differ by functional status and lineage [17]. For instance, functional PitNETs are more likely than nonfunctional tumors to harbor somatic copy number alterations (SCNAs), with some tumors exhibiting large-scale chromosomal involvement affecting up to 99% of the genome [18]. The most frequently observed chromosomal changes include losses of chromosomes 1p and 11, suggesting that mitotic errors, rather than focal driver events, may underlie much of the genomic disruption in these tumors. These copy number variations (CNVs) are not uniformly distributed across PitNET subtypes and have been associated with both biological behavior and clinical outcomes. Lactotroph tumors, for example, tend to carry a higher CNV burden than corticotrophs, somatotrophs, gonadotrophs, or immunonegative tumors, a pattern that has been linked to poorer prognosis, even when controlling for other risk factors such as invasion and proliferation [19]. These findings support the view that tumor subtype, while informed by TF expression, may be further stratified by specific molecular alterations that shape behavior, progression, and therapeutic response (Fig. 2). The sections below explore these subtype-specific molecular profiles in greater detail.

### Prolactinomas

As with most pituitary tumors, the vast majority (90–95%) of prolactinomas harbor somatic rather than germline mutations. A somatic mutation in the splicing factor 3 subunit B1 (*SF3B1*) has been described in roughly 20% of prolactinomas and has been associated with increased prolactin secretion (via aberrant estrogen receptor splicing and increased PIT1 binding) and shorter progression free survival in patients [20]. In mice, *SF3B1* mutations have been shown to stimulate PI3K/AKT signaling via downregulation of the tumor suppressor gene Discs large 1 (DLG1), resulting in increased prolactinoma invasion and migration [21]. 

MicroRNA (miRNA) deregulation has also been described in sporadic prolactinomas. The miRNA miR-183, which directly targets KIAA0101 (an inhibitor of p53–p21-mediated cell cycle arrest), is specifically down-regulated in aggressive prolactinomas, resulting in S phase inhibition and cell proliferation [22]. Derangements at the protein level have also been described in prolactinomas. HMGA proteins - TFs expressed during embryogenesis and frequently overexpressed in neoplasms [23] – have been implicated in prolactinoma pathogenesis. Amplification of HMGA2 through gain of chromosome 12 has been demonstrated in 89% of prolactinomas and may contribute to resistance to dopaminergic therapies [24]. Additionally, HMGA2 interacts with the cell cycle regulators p27^kip1^ and cyclin-dependent kinase 4 (CDK4) in mouse models to promote tumorigenesis, with increased incidence of pituitary PitNETs with faster and more invasive growth and high Ki67 index (a marker of cell proliferation and mitotic activity) [25]. 

Epidermal growth factor receptor (EGFR) expression occurs in over 80% of prolactinomas, with expression level associated with tumor aggressiveness [26]. High levels of EGFR have been correlated with increased prolactinoma invasion and reduced potential for gross total resection [27]. A broad distribution of EGFR binding sites has been described specifically in prolactinomas, with EGFR-positive cells observed more frequently than non-prolactinoma tumor tissue [28]. In vitro studies of an EGFR-targeting micro-RNA, mir-137, and Osimertinib (a tyrosine kinase inhibitor (TKI)), demonstrates inhibition of cell proliferation and migration as well as cell cycle arrest [27]. In mouse models, the EGFR inhibitor gefitinib blocks prolactin gene expression, decreases prolactin secretion, and controls tumor growth via decreased extracellular signal-regulated kinase (ERK)1/2 phosphorylation [29].

A minority of prolactinomas arise from germline mutations as part of familial syndromes. In Multiple Endocrine Neoplasia type 1 (MEN1), prolactinomas are the most common pituitary tumor phenotype, representing 60% of pituitary tumors occurring in MEN1 patients [30]. In Familial Isolated Pituitary Adenoma (FIPA)—a condition most often associated with pathogenic variants in the aryl hydrocarbon receptor–interacting protein (*AIP*) gene—approximately 40% of affected individuals develop prolactinomas [31]. Other rare hereditary conditions that can present with prolactinomas more frequently than the general population include MEN4, Carney complex, and McCune–Albright syndrome, though these remain uncommon overall [32, 33].

### Gonadotroph PitNETs

Nearly all gonadotroph PitNETs are sporadic rather than part of a familial syndrome. These tumors are typically silent, presenting as clinically nonfunctioning macroadenomas with low proliferative indices and minimal genomic alterations, including low copy number variations (CNV) with a largely stable genome [19]. Despite this innocuous molecular and biochemical profile, gonadotroph PitNETs often present as large, invasive macroadenomas, although this may reflect slow growth before reaching a size causing symptomatic mass effect due to their nonsecretory nature [34], as gonadotroph PitNETs are rarely classified as aggressive based on growth seen on serial imaging. Activation of developmental signaling pathways, including the Wnt/β catenin pathway, can be seen in gonadotroph PitNETs, particularly those with more invasive phenotypes [35]. 

### Somatotroph PitNETs

Somatotroph PitNETs are characterized by excess GH secretion and typically exhibit low CNVs with a largely stable genome [19]. Most familial and sporadic mutations in somatotroph PitNETs are associated with protein kinase A (PKA) activation [36]. These mutations often activate PKA via adenylate cyclase, the enzyme activated when Growth Hormone Releasing Hormone (GHRH) drives GH production by normal somatotroph pituitary cells. Adenylate cyclase generates cAMP, which in turn activates PKA, which travels to the cell nucleus where it phosphorylates a TF called cAMP response element-binding protein (CREB). Phosphorylated CREB then binds to a specific DNA promoter region, leading to the increased expression of the pituitary-specific TF, PIT1, which in turn increases GH transcription and somatotroph cell proliferation.

Less than 5% of somatotroph PitNETs are associated with familial syndromes [37]. FIPA, which is caused by germline loss-of-function mutations in the *AIP* gene, drives somatotroph tumorigenesis by disrupting cAMP/PKA regulation and accounts for 20–40% of these familial acromegaly cases, with a tendency to give rise to early-onset, aggressive somatotroph PitNETs with poor response to somatostatin analogues (SSAs) [38]. Functional variants of *CDH23*, which encodes an adhesion molecule, have been identified in around 33% of germline somatotroph PitNETs, sometimes co-occurring alongside *AIP* mutations in families with FIPA [39]. While proof of the role of *CDH23* alterations in somatotroph tumorgenesis or growth has yet to be established, the finding that these variants are associated with increased tumor invasiveness despite smaller tumor size offering intriguing clues as to the functionality of these variants [40]. In patients with *MEN1* mutations, somatotroph PitNETs are the second most common pituitary tumor, representing 20% of the PitNETs diagnosed in these patients. Duplications of the *GPR101* gene, which encodes an orphan G protein-coupled receptor (GPCR), are a major cause of X-linked acrogigantism (XLAG) [41]. Inactivating mutations in the *PRKAR1A* gene, which encodes the regulatory subunit (R1α) of protein kinase A (PKA), occur in less than 1% of familial acromegaly cases as part of Carney Complex (CNC), a rare genetic disorder in which 10–12% of affected patients develop acromegaly [42]. 

Over 95% of somatotroph PitNETs harbor sporadic mutations, with the most common example being mutations in the *GNAS* gene, which encodes a G-protein. *GNAS* mutations occur in 30–40% of sporadic somatotroph PitNETs [43]. If the *GNAS* mutation affects just the pituitary, then the somatotroph PitNET will be its only manifestation, but some patients develop embryonic *GNAS* mutations leading to mosaicism, a non-hereditary (due to its not involving the germline) condition known as McCune Albright syndrome in which 20–30% of patients develop acromegaly and patients can also develop fibrous dysplasia or café-au-lait spots [44]. Most of the documented *GNAS* mutations in somatotroph PitNETs are activating mutations in codons 201 or 227 and lead to constitutive activation of cAMP/PKA signaling, thereby bypassing normal regulatory controls and leading to chronic overproduction of GH and clinical acromegaly [40, 45]. Somatotroph PitNETs with *GNAS* mutations tend to be smaller and more responsive to somatostatin analogs than somatotroph PitNETs without *GNAS* mutations [46]. Mutations in the *AIP* gene occur in 3–4% of sporadic somatotroph PitNETs [47]. Duplications of the *GPR101* gene are the primary pathogenic defect found in 8–10% of patients with non-syndromic pituitary gigantism [41, 48]. Nearly 50% of childhood-onset cases of acromegaly have an identifiable genetic cause, with *GPR101* being one of the most common, after *AIP* mutations. Lastly, functional variants of *CDH23* have been identified in 12% of sporadic somatotroph PitNETs [39].

### TSH-secreting tumors

Thyrotropin secreting pituitary tumors account for approximately 1–2% of PitNETs and are almost always sporadic rather than familial in nature [49]. Mutations in the TSH receptor have been identified in these tumors, with overexpression of the TRβ subunit noted in multiple surgical specimens [50–52]. Alternative splicing of TRβ2 mRNA has been shown to generate an abnormal thyroid receptor (TR) protein, resulting in defective negative regulation of TSH [53]. Mouse models exhibit spontaneous development of thyrotroph tumors in knock-in mice bearing TRβ mutations, with gene profiling of these tumors demonstrating constitutive activation and overexpression of cyclin D1 (a cell cycle regulator and known proto-oncogene) [54]. TSH dysregulation in thyrotroph tumors may also be influenced by deiodinase enzymes, which metabolize thyroid hormone and determine tissue concentrations of the biologically active ligand. One study demonstrated a 13.1-fold excess of deiodinase enzyme mRNAs in thyrotropic tumor tissue compared to normal pituitary tissue [55]. 

Mutated GATA2, a TF found to be critical for the differentiation and maintenance of thyrotroph cells in an in vivo mouse model [56], has been identified in thyrotroph PitNETs [57] GATA2 is believed to contribute to tumorigenesis by regulating TSHβ gene transcription, either directly or via interaction with other pituitary-specific TFs such as PIT1 [58, 59]. GATA2 also maintains type 2 deiodinase (D2) expression in thyrotrophs, which converts the prohormone thyroxine (T4) into the active hormone triiodothyronine (T3), and helps to maintain euthyroid homeostasis via tight regulation of TSH secretion via T3 feedback loops [60]. Elevated expression of GATA2 has been observed in some thyrotroph tumors [57], and GATA2’s critical role in maintaining TSHβ and D2 expression suggests that dysregulation—such as overexpression or loss of negative feedback—could contribute to their hypersecretory phenotype.

### Corticotroph tumors

Corticotroph tumors are pituitary tumors arising from ACTH-secreting cells, and the hypersecreting phenotype is the most common cause of Cushing’s disease. TPIT is a lineage-defining TF for corticotroph tumors, and these tumors are divided into three classes: (1) *USP8-*wild-type (most aggressive), (2) *USP8*-mutated (more overt secretion, better response to therapy), and (3) large silent tumors with gonadotroph transdifferentiation [16]. Ubiquitin specific peptidase 8 (*USP8*) mutations are found in approximately 20–60% of corticotroph tumors and are typically somatic gain-of-function mutations [61]. *USP8* removes ubiquitin tags from targets, such as Smoothened (*SMO*) and EGFR, preventing their degradation. Mutations in *USP8* impair protein binding, lead to increased *USP8* activity which enhances stabilization and activation of the *SMO* and EGFR transmembrane proteins. *SMO* and EGFR drive POMC production through pathways outside of the cAMP-driven production that normal corticotroph cells rely on. *SMO* activates the hedgehog (Hh) signaling pathway, which prevents GLI TFs (specifically GLI2 and GLI3) from being repressed by the suppressor of fused protein (SUFU). The GLI activators then translocate to the nucleus, where they drive *POMC* gene expression [62]. EGFR activates the MAPK/ERK pathway, leading to phosphorylation of the E2F1 TF, which drives *POMC* gene expression [63]. Corticotroph tumors have also been shown to have significantly more EGFR expressing cells than other functional PitNET subtypes [64]. One study demonstrated overexpression of EGFR in 55.8% of pituitary corticotroph tumors, and EGFR expression levels were positively associated with the ACTH and cortisol levels and with tumor recurrence [65]. Targeting EGFR in animal models with Cushing’s Disease has been shown to decrease tumor size and cortisol levels [66–68]. 

Other gene mutations described in corticotroph tumors include *USP48*, a ubiquitinase smilar to *USP8* mutated in 35–62% of corticotroph tumors [69]; *BRAF*, which encodes a serine/threonine kinase and is mutated in 9–19% of corticotroph tumors [69]; *ATRX*, which is mutated in 20–30% of corticotroph tumors that are particularly aggressive [70]; and *TP53*, which is mutated in 12% of corticotroph tumors, often in larger, more aggressive *USP8*-wildtype macroadenomas [71]. Similar to *USP8* mutants, both *USP48* and *BRAF* mutants enhance the promoter activity and transcription of *POMC* [69]. In contrast, *ATRX* and *TP53* mutations lead to dysregulated corticotroph cell proliferation, with an associated increase in net ACTH production due to this proliferation. Less than 5% of corticotroph tumors arise as part of familial syndromes, with cases reported as part of Multiple Endocrine Neoplasia type 1 (MEN1), Carney complex, or familial isolated pituitary adenoma (FIPA) syndrome [37]. 

Silent corticotrophic adenomas (SCAs) also arise from ACTH-producing cells but differ dramatically in clinical behavior, hormonal activity, and molecular features. While POMC transcription is preserved in SCAs relative to hypersecreting corticotroph PitNETs, expression of the PCSK1 (the gene encoding the enzyme PC1/3 which cleaves POMC), is markedly reduced, leading to an accumulation of unprocessed POMC that is detected when the tumor is immunostained for ACTH, but insufficient production of active ACTH [72]. This loss of processing of POMC into hormonally active ACTH is not the only molecular feature that distinguishes SCAs from hypersecreting corticotroph PitNETs. By single-cell RNA sequencing, SCAs demonstrate higher expression of chromogranin A and secretogranin II (seen in nonfunctioning tumors), and upregulation of genes involved in epithelial-to-mesenchymal transition (EMT) and cell migration, suggesting a more invasive phenotype [16]. In contrast to the hypersecreting PitNETs, SCAs rarely harbor *USP8* mutations [61]. 

### Null cell tumors

Null cell tumors are a subtype of PitNETs that lack both hormonal secretion and expression of lineage-defining TFs; they represent less than 5% of PitNETs. Molecularly, they exhibit a low hormone gene expression signature, variable epigenetic patterns, and a heterogeneous transcriptome, suggesting either arrested differentiation or origin from non-committed progenitor cells [73]. 

Null cell tumors are almost always sporadic and are primarily comprised of chromophobic cells, which display a negative PAS reaction indicating a lack of secretory granules, which may represent a less differentiated state than other PitNETs [73]. Overexpression of HIPPO pathway components (i.e. large tumor suppressor homolog 2, LATS2, and TEAD4) have been implicated in null cell tumors [74]. The Hippo pathway regulates cell proliferation and apoptosis by inhibiting the nuclear activity of the transcriptional coactivators YAP and TAZ [75]. Null cell tumors demonstrate increased nuclear localization YAP/TAZ, activation of which represses pituitary cell differentiation, contributing to the null phenotype [74]. YAP/TAZ have also been shown to co-localize with SOX2-positive pituitary stem cells, leading to expansion of stem/progenitor cells, blockage of differentiation, and formation of nonfunctioning and aggressive pituitary lesions [76]. Some cohorts of null cell tumors also exhibit subtype specific mutations in the chromatin modifier *ASXL1* as well as the proto-oncogene neurotrophic receptor tyrosine kinase 1 (*NTRK1*), which encodes the receptor for nerve growth factor (NGF) and plays a crucial role in regulating neuronal survival, differentiation, and proliferation [77]. These mutations may promote tumorigenesis by disrupting the epigenetic control of differentiation and proliferation, and by sustaining growth and resistance to differentiation, respectively.

## Molecular markers of aggressive PitNETs

At present, the most reliable way to classify clinical aggressiveness of PitNETs comes from cases which have unusually rapid growth documented on two sets of images over time, with aggressive PitNETs defined as a ≥ 20% increase in maximum tumor diameter over 6–12 months [78]. PitNETs meeting this criteria of aggressiveness are associated with invasiveness, resistance to therapy, and frequent recurrence [79]. Aggressive PitNETs are predominantly reported among lactotroph and corticotroph subtypes [79].

A range of molecular markers have been associated with the aggressive PitNET phenotype (Table 1), including those regulating proliferation and cell cycle regulation, invasion and extracellular matrix remodeling, DNA repair, stemness and growth, and immune response. Key proliferative and genomic instability markers include Pituitary Tumor-Transforming Gene (*PTTG*), which promotes chromosome missegregation and tumor invasiveness [80], and mutant *TP53*, which has been linked to increased mitotic index, poor prognosis, and high-grade transformation in both PitNETs and many other cancers [81]. *EZH2*, a histone methyltransferase and one of the most consistently upregulated epigenetic regulators in aggressive PitNETs, correlates with increased proliferation (e.g., high Ki-67 index), increased invasiveness, and EMT-related gene expression in both functioning and non-functioning PitNETs [82]. Disruption of DNA repair pathways—such as low MGMT expression and loss of MSH6/MSH2—further contributes to genomic instability and therapeutic resistance, particularly to alkylating agents like temozolomide [83]. Markers involved in extracellular matrix remodeling and local invasion, such as MMP-2, MMP-9, MMP-14, and Galectin-3 [84], are upregulated in aggressive tumors, facilitate tissue infiltration, correlate with cavernous sinus invasion and recurrence [85]. 

**Table 1:** Summary of molecular markers of aggressive tumors

Marker	Function	Aggressive Behavior	Associated PitNET Subtype(s)
PTTG	Chromosome separation, cell cycle control	Invasion, recurrence, signal of aggressiveness	Multiple (esp. corticotroph, somatotroph, gonadotroph)
p53 (mutant)	DNA repair/apoptosis regulation	Higher grade, invasion, poor prognosis	Multiple (esp. corticotroph and silent subtypes)
EZH2	Histone methylation	Correlates with invasion, high Ki-67	Multiple (esp. non-functioning)
MMP-2/-9/-14	ECM degradation	Cavernous sinus invasion, tumor infiltration	Multiple (esp. gonadotroph, corticotroph, silent subtypes)
Galectin-3	Cell proliferation, anti-apoptosis	Invasion, recurrence, decreased drug response	Lactotroph and non-functioning adenomas
MGMT (low expression)	DNA repair deficiency	TMZ sensitivity, but also aggressive/recurrent features	Corticotroph and plurihormonal tumors
MSH6 / MSH2	Mismatch repair	Genomic instability, disease progression	Corticotroph
EMT factors	Promote cell motility/invasion	Invasive phenotype, ECM remodeling	Primarily non-functioning and plurihormonal tumors
HMGA1 / HMGA2	Chromatin architecture	Invasion, proliferation, GH-secreting tumor aggressiveness	GH-secreting (somatotroph) and non-functioning tumors
DPPA4 / Wnt	Stemness, migration, proliferation	Aggressive markers in vitro and ex vivo	Non-functioning PitNETs with stemness-like features
PD-L1	Immune checkpoint	Proliferation, p53 positivity, immune escape potential	Somatotrophs and corticotrophs
ATRX	Chromatin remodeling	High mitotic rates, elevated Ki-67, and resistance to standard therapies	Silent corticotroph adenomas, poorly differentiated tumors, pituitary carcinomas

A growing body of evidence also highlights the importance of epigenetic and stemness-associated genes in driving the genetic plasticity and progression of aggressive PitNETs. Overexpression of HMGA1 and HMGA2, which modulate chromatin architecture, has been linked to increased proliferation and invasiveness in somatotroph and plurihormonal tumors [86]. Similarly, activation of DPPA4 and Wnt/EMT-related pathways (including Snail and ZEB) supports the emergence of stem-like, migratory phenotypes, often in non-functioning tumors that lack clear hormonal identity [87]. Loss of ATRX, a chromatin remodeler essential for telomere maintenance, marks a crucial transition to a more genetically unstable and treatment-resistant state in aggressive PitNETs, particularly silent corticotrophs and pituitary carcinomas [88]. Finally, PD-L1 expression may facilitate immune evasion in aggressive lactotroph and corticotroph PitNETs, suggesting its potential relevance as a therapeutic target [89]. Together, these markers reflect diverse biological mechanisms driving PitNET aggressiveness and provide a foundation for improved risk stratification and targeted treatment approaches.

Metastasis outside of the sellar and suprasellar region is a rare PitNET phenotype, occurring in fewer than 1% of PitNETs, and is associated with a poor prognosis and a need for adjuvant chemotherapy and radiation treatment [90]. While metastatic PitNET cases may not always have the serial imaging needed to characterize them as aggressive PitNETs, they share a number of the epigenetic and genetic changes found in aggressive PitNETs [91].

The failure of surgery and radiation to control the growth of aggressive PitNETs has led to efforts to leverage advances in the molecular characterization of PitNETs into new avenues for clinical trials of molecularly targeted therapies (Table 2), particularly for patients with aggressive or treatment-resistant tumors that are secretory in nature, as secretory tumors tend to have more targetable molecular alterations and require treatment in the setting of residual tumor after surgery due to the effects of their hypersecretion. EGFR inhibition has been trialed in patients with aggressive or dopamine agonist-resistant prolactinomas. A small trial of 2 patients treated with a 6-month course of the oral EGFR/HER2 TKI Lapatinib demonstrated normalization of prolactin level and a 22% reduction in tumor volume in one patient, and stable disease with 42% decrease in prolactin level in the other [92]. A subsequent phase IIa trial of Lapatinib demonstrated stable disease for 75% of participants, with one patient exhibiting a 16.8% decrease in tumor diameter, though prolactin suppression was not consistently observed [93]. Given that somatic *USP8* mutations enhance EGFR signaling and can drive excess ACTH secretion in corticotroph PitNETs, tumors harboring these mutations have also been specifically targeted with EGFR inhibitors. A phase II trial of Gefitinib, an EGFR inhibitor FDA approved in non-small cell lung cancer, is ongoing for treatment of *USP8*-mutated corticotrophin adenomas (NCT02484755). Primary endpoints include reduction of serum ACTH and urinary free cortisol levels, though no preliminary data has been published to date [94]. 

**Table 2:** Targeted clinical trials for pituitary adenomas

Trial ID	Phase	Agent	Target	Tumor Subtype	No. Patients Treated	Status	Results
Investigator-Initiated Single Cohort (Cedars-Sinai IRB #18129)	Phase 2	Lapatinib	EGFR/HER2	Prolactinoma (Dopamine Resistant)	2	Closed	22% tumor reduction (1), stable disease (1); prolactin reduction (1) or normalization (1)
NCT00939523	Phase 2a	Lapatinib	EGFR/HER2	Prolactinoma (Dopamine Resistant)	4	Closed	Stable disease (3), 16.8% tumor reduction (1); no significant prolactin suppression
NCT02484755	Phase 2	Gefitinib	EGFR	Corticotroph Adenoma (USP8-mutated)	Unknown	Unknown	Trial results not published to date
NCT02160730/ NCT03774446	Phase 2	Seliciclib (R-roscovitine)	CDK	Corticotroph Adenoma with Cushing’s Disease (de novo or recurrent)	9	Closed	42% reduction in mean urine free cortisol; >50% reduction (3), 48% reduction (2); 19% reduction in plasma ACTH in UFC responders
NCT04042753	Phase 2	Nivolumab + Ipilimumab	PD-1/CTLA-4	Pituitary Adenoma/Carcinoma (any histology)	Unknown	Active	Trial results not published to date; case report of off-label use in hypermutated ACTH-secreting carcinoma with complete response and no residual tumor burden at 34 months
NCT00088582	Phase 2	Pasireotide	SST1-3, SST5	GH-Secreting Adenoma	60	Closed	27% of patients with reduction in GH and normalization of IGF-1 at 3 months; 39% of patients with > 20% reduction in tumor volume
NCT01374906	Phase 3	Pasireotide	SST1-3, SST5	Corticotroph Adenoma with Cushing’s Disease (de novo or recurrent)	150	Closed	40% of patients with normalization of UFC at 7 months
NCT05192382	Phase 3	Paltusotine	SSTR2	GH-Secreting Adenoma	111	Closed	55.6% of paltusotine-treated patients with IGF-I ≤ 1.0 ×ULN vs. 5.3% for placebo; 92.6% of patients with reduction in IGF-1 at end of treatment
NL5136 (Netherlands)	Phase 3	Lanreotide	SST2, SST5	SSTR + Non-Functioning Pituitary Macroadenoma	32	Closed	No reduction in tumour size or growth compared to placebo
NCT04522180	Phase 2	IONIS-GHR-LRx (Cimdelirsen)	GH Receptor	GH-Secreting Adenoma	43	Closed	Dose dependent reduction in IGF-1, GHBP, and IGFBP3

Other targeted approaches have been trialed for ACTH secreting tumors, including a phase II trial of the CDK inhibitor seliciclib (R-roscovitine) with preliminary findings showing reduced plasma ACTH levels and urine free cortisol (UFC) in a subset of patients [95]. Immunotherapy with the combination of nivolumab/ipilimumab is also actively under investigation for patients with Cushing’s Disease. While formal trial data are pending, a published case report of a hypermutated ACTH-secreting carcinoma treated off-label with this combination showed marked tumor regression [96], offering a rationale for checkpoint blockade in select molecular contexts.

Somatostatin receptors (SSRs) have long been targeted via somatostatin analogues (SSAs) for residual somatotroph PitNETs after surgery. SSAs have also been used off-label to treat aggressive non-somatotroph PitNETs with limited treatment option because these agents have potential to slow the growth of SSR-expressing non-somatotroph PitNETs, although those tumors are typically less reliant on SSRs for their growth than somatotroph PitNETs [97]. SSAs have historically included established long-acting injectable forms like octreotide, which binds SSR2, SSR3, and SSR5; lanreotide, which binds SSR2 and SSR5; and pasireotide, a more recent addition to the family which is appealing due to its higher affinity and ability to bind more receptors: SSR1, SSR2, SSR3, and SSR5 [98]. Pasireotide is approved in the United States for patients with Cushing’s disease or acromegaly in which surgery has failed, or surgery is not possible, or for those who have acromegaly for which other SSAs have failed to achieve biochemical remission [99]. A long-acting form of pasireotide also achieved normalization of UFC in 40% of patients with corticotroph PitNETs [100]. The most significant recent advance in SSAs has been the development of oral forms such as the nonpeptide highly selective SSR2 agonist Paltusotine, which has biochemical results in acromegaly comparable to standard-of-care injectable therapy [101]. While SSAs targeting SSR2 were felt to be best suited for treating aggressive non-functional PitNETs due to SSR2 being the most common SSR expressed by these tumors, the recently completed GALANT trial which enrolled nonfunctional macroadenomas that were positive on PET using the SSR2-specific tracer ^68^Ga-DOTATATE found no significant benefit of lanreotide, which has SSR2 and SSR5 affinity, in controlling the size of these tumors, calling into question the role of SSAs in treating these tumors [102]. SSR treatments have also been combined with potentially synergistic therapeutic strategies. A Phase II trial of adjunctive IONIS‑GHR‑LRx (cimdelirsen) – an antisense oligonucleotide targeting GH receptor – in patients with acromegaly resistant to long-acting SSAs demonstrated significant reduction in IGF-1 with a concurrent drop in GH-binding protein (GHBP) and IGF-binding protein 3 (IGFBP3) levels [103].

Trials for drugs targeting somatostatin and growth hormone receptors have shown promising results in patients; however, given the number of patients who are unable to undergo surgery and for whom octreotide and other somatostatin analogues do not work, the small size of this group makes it unclear just how much clinical utility these drugs offers in the current therapeutic landscape. Collectively, these trials represent a shift toward individualized management strategies in PitNETs—leveraging molecular diagnostics, signaling pathway inhibition, and immune modulation—tailored to tumor subtype, biological behavior, and genetic context. While early-phase efficacy signals are promising, larger, stratified trials are essential to validate and integrate these molecularly guided approaches into clinical practice.

## Discussion and future directions

The current pituitary tumor classification scheme based on the expression of lineage-specific TFs—TPIT, PIT1, and SF1—reflects the embryologic origins of hormone-producing cell types within the anterior pituitary. While biologically intuitive and useful for distinguishing tumor subtypes, this framework provides limited insight into tumor behavior, particularly regarding aggressiveness, recurrence risk, or treatment responsiveness. It is increasingly clear that this lineage-based taxonomy does not fully capture the molecular heterogeneity or clinical heterogeneity of pituitary neuroendocrine tumors. As such, several alternative classification schemes that integrate clinical, genetic, biochemical, radiological, pathological, and molecular information have been proposed [104–106] which will need to be prospectively validated in order to ensure the development of a classification scheme that is broadly useful to both pathologists and clinicians caring for pituitary tumor patients.

A further question concerns the cell of origin of pituitary tumors. While the current model assumes that tumors arise from lineage-committed progenitors of PIT1, TPIT, or SF1, the detection of pluripotent stem-like cells expressing markers in some tumors raises the possibility that a common pituitary progenitor or stem-like cell may underlie multiple tumor subtypes. This hypothesis is supported by reports of tumors expressing multiple lineage markers, suggesting a degree of plasticity in pituitary tumor development. Further investigation into stem cell–related transcriptional programs and single-cell transcriptomics will be key to resolving this question.

Additionally, the inclusion of the term “neuroendocrine” in the 2022 WHO classification reflects the anatomical and developmental origins of pituitary cells but remains a source of debate. Unlike other neuroendocrine tumors, PitNETs typically lack well-defined neuroendocrine markers such as chromogranin A, and rarely metastasize. Whether they should be considered within the broader NET spectrum or as a unique class of endocrine tumors remains to be resolved. Addressing this issue will require comparative molecular profiling and clarity on the shared versus divergent epigenetic and transcriptomic landscapes of pituitary and extra-pituitary neuroendocrine tumors.

One of the most recurrent genomic findings across PitNETs is the presence of large-scale chromosomal losses or gains. Frequent losses involving chromosomes 1p, 11q, and 13q have been reported in multiple tumor types, including null cell, gonadotroph, and somatotroph PitNETs. Yet, the oncogenic relevance of these alterations remains poorly defined. Are these events early tumorigenic drivers, markers of genomic instability, or consequences of disrupted cell cycle regulation? Without more functional data, these copy number variations (CNVs) remain molecular signposts with uncertain mechanistic value. Another critical unknown is whether aggressive PitNETs arise through stepwise accumulation of genetic or epigenetic changes in otherwise indolent tumors (i.e., the “second hit” model), or whether they are biologically distinct from the outset. Mutations in genes such as ATRX, p53, and PTTG, as well as overexpression of EZH2 and stemness markers, have been associated with more aggressive or clinically refractory tumors. However, it remains unclear whether these features are acquired during progression or reflect early divergence in tumor cell fate. Answering this question has direct clinical implications for surveillance strategies and treatment in high-risk subgroups.

Moving forward, the integration of multi-omics data, including whole-genome sequencing, epigenetic mapping, proteomics, and spatial transcriptomics, will be essential to move beyond simplistic classifications and toward a biologically grounded framework that incorporates molecular, cellular, and clinical elements. Identifying reliable biomarkers for tumor progression and molecular vulnerabilities for targeted therapy should remain a priority in order to translate our growing molecular knowledge into meaningful clinical advances for patients with both benign and aggressive PitNETs.

## Data Availability

No datasets were generated or analysed during the current study.
